# Individual‐level brain morphological similarity networks: Current methodologies and applications

**DOI:** 10.1111/cns.14384

**Published:** 2023-07-30

**Authors:** Mengjing Cai, Juanwei Ma, Zirui Wang, Yao Zhao, Yijing Zhang, He Wang, Hui Xue, Yayuan Chen, Yujie Zhang, Chunyang Wang, Qiyu Zhao, Kaizhong Xue, Feng Liu

**Affiliations:** ^1^ Department of Radiology and Tianjin Key Laboratory of Functional Imaging Tianjin Medical University General Hospital Tianjin China; ^2^ Department of Scientific Research Tianjin Medical University General Hospital Tianjin China

**Keywords:** brain network, individual level, morphological similarity network, structural magnetic resonance imaging

## Abstract

**Aims:**

The human brain is an extremely complex system in which neurons, clusters of neurons, or regions are connected to form a complex network. With the development of neuroimaging techniques, magnetic resonance imaging (MRI)‐based brain networks play a key role in our understanding of the intricate architecture of human brain. Among them, the structural MRI‐based brain morphological network approach has attracted increasing attention due to the advantages in data acquisition, image quality, and in revealing the structural organizing principles intrinsic to the brain. This review is to summarize the methodology and related applications of individual‐level morphological networks.

**Background:**

There have been a growing number of studies related to brain morphological similarity networks. Conventional morphological networks are intersubject covariance networks constructed using a certain morphological indicator of a group of subjects; individual‐level morphological networks, on the other hand, measure the morphological similarity between brain regions for individual brains and can reflect the morphological information of single subjects. In recent years, individual morphological networks have demonstrated significant worth in exploring the topological changes of the human brain under both normal and disease conditions. Such studies provided novel perspectives for understanding human brain development and exploring the pathological mechanisms of neuropsychiatric disorders.

**Conclusion:**

This paper mainly focuses on the studies of brain morphological networks at the individual level, introduces several ways for network construction, reviews representative work in this field, and finally points out current problems and future directions.

## Graphical abstract

The construction of individual‐level brain morphological similarity networks. (A) The basis for constructing individual‐level morphological brain networks: morphological metrics extracted from individual structural MRI. (B) The single metric‐based methods for constructing individual morphological similarity networks. (C) The multiple metrics‐based methods for constructing morphological similarity networks. (D) Each of the above methods ultimately generates the similarity matrix, which is subsequently used to generate the network graphs for further graph‐theoretic analyses.

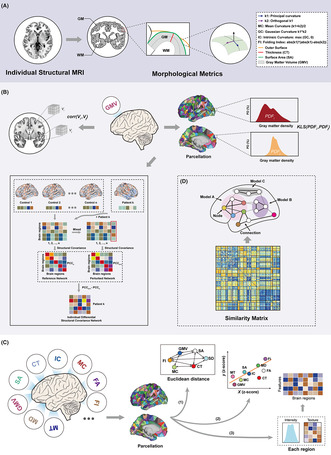

## 1 BACKGROUND

The human brain is a highly integrated network rather than an isolated anatomical area, and it forms a complex and precise connectivity framework through synaptic interactions between neurons.^1^ Numerous brain science‐related studies have shown that the execution of various higher level cognitive functions of the brain relies on the synergistic cooperation of spatially distributed brain regions, instead of just one specific region.^2^ To some extent, the pathogenesis of many neuropsychiatric disorders (e.g., schizophrenia and depression) is thereby attributed to the dysconnectivity between the brain regions involved.^3^, ^4^, ^5^

**FIGURE 1:**
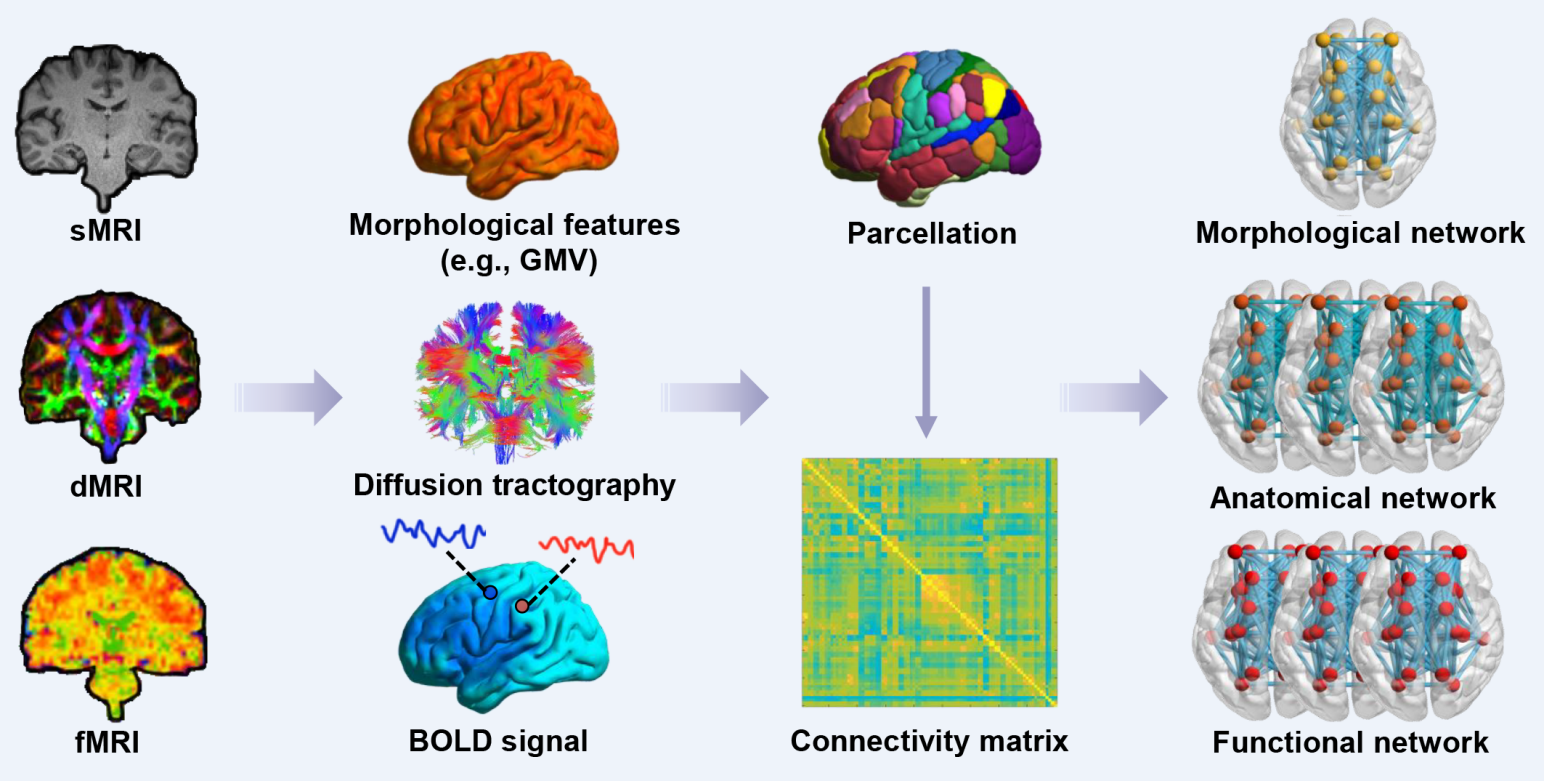
A flowchart for constructing brain morphological, anatomical, and functional networks. The construction of human brain morphological, anatomical, and functional networks involves the utilization of sMRI, dMRI, and fMRI data, encompassing the following procedures: defining network nodes using a priori atlas to parcellate the whole brain, defining network edges of estimating relationship between nodes (morphological connectivity is defined as the interregional similarity of morphological features, anatomical connectivity is based on diffusion tractography, and functional connectivity is generally defined as the statistical correlation of BOLD signals across different regions), obtaining connectivity matrix and finally generating the morphological network (group level), anatomical as well as functional network (individual level). BOLD, blood oxygen level dependent; dMRI, diffusion magnetic resonance imaging; fMRI, functional magnetic resonance imaging; GMV, gray matter volume; sMRI, structural magnetic resonance imaging.

Over the past few decades, neuroscientists have been fully aware of the importance of exploring human brain connectivity patterns and formally proposed the concept of human connectome to draw attention to this topic.^6^ The human connectome seeks to map the brain connectivity network from microscale (single neurons and synapses), mesoscale (neuronal clusters), and macroscale (brain regions) as well as to reveal the potential mechanisms of brain network functioning in normal or diseased states. The development of modern imaging methodology has given essential tools to the exploration of brain connectome. Currently, research in this field is mainly concentrated on the macroscale level, with the aim of constructing brain networks through various magnetic resonance imaging (MRI)‐based neuroimaging techniques, which include functional MRI (fMRI)‐based functional networks,^7^, ^8^ diffusion MRI (dMRI)‐based anatomical networks,^9^, ^10^ and structural MRI (sMRI)‐based morphological networks^11^ (Figure 1). The brain functional network describes the synergistic cooperation of different brain regions, anatomical network reflects interregional fiber connectivity, and morphological network portrays interregional morphological similarity. Once structural (i.e., anatomical/morphological) connectivity or functional connectivity has been mapped for the brain, the topological properties and internal working mechanisms of the brain can be further revealed by combining complex network analysis in terms of graph theory.^2^, ^12^

Notable progress has been made in the field of brain functional connectome and dMRI‐based anatomical connectome, yet relevant research on morphological brain networks started relatively late. Compared with fMRI and dMRI data, sMRI is relatively low cost, easier to use for large‐scale studies, and has more consistent image quality. In addition, the sMRI data can provide more accurate information about brain structures, and several studies have found that the morphological data of the human brain contains a large amount of brain connectivity information.^13^ In 2005, Mechelli et al.^14^ identified significant gray matter (GM) density covariance between certain regions of the human brain by analyzing sMRI data, and it was speculated that this coordination may be related to the white matter tracts (corpus callosum) connections between them. A later exploration by Lerch et al.^15^ assessed the interregional statistical correlation in cortical thickness within a population sample. Using Brodmann area 44 as the seed region, they found that the cortical thickness correlation maps were strikingly similar to the tractography maps of arcuate fasciculus obtained from dMRI, suggesting that the morphological correlation may have parallels with brain anatomical connectivity. Therefore, the sMRI‐based network approach is a promising tool for the study of structural brain connectome.

The morphological networks can be divided into intersubject (i.e., group‐level) covariance networks and individual‐level brain morphological similarity networks depending on how they are constructed. In graph‐theoretic models, the network nodes are generally defined using brain regions parcellated according to a priori atlas, while the edges represent morphological connectivity between brain regions. Intersubject covariance networks, as the name suggests, are constructed by calculating interregional statistical correlations of a certain morphometric feature (e.g., cortical thickness, surface area, or GM volume) across a group of subjects; in contrast, individual morphological networks are constructed by measuring interregional morphological similarities within a single subject. Early construction of morphological networks was usually at the group level. In 2007, He et al.^11^ successfully constructed the first brain morphological network using cortical thickness measurements from a cohort of subjects. In conjunction with the graph‐theoretic model, they confirmed the small‐world properties and degree distribution in large‐scale morphological networks, and these findings were largely consistent with previous functional networks studies.^16^, ^17^ In the wake of this, many researchers followed the idea raised by He et al. and carried out further work related to intersubject covariance networks. For example, Chen et al.^18^ identified a modular organization pattern of the network derived from cortical thickness measurement with each module corresponding to known functional regions (e.g., language, memory, and vision); Diaz et al.^19^ demonstrated that cortical surface area was a useful indicator for studying morphological connectivity networks.

Aforementioned sMRI‐based studies showed that the morphological network has topological properties such as small‐worldness and modular architecture, which provides a simple and effective way to describe the connectivity patterns of the human brain. To date, the population‐based morphological covariance network methods have been used as an important tool to study the human brain. Nevertheless, these methods can only construct one network for a whole group of subjects, thereby missing morphological information of individual brains. This limits its application in the study of individual variability in brain structure, particularly in identifying morphological abnormalities in the patient's brain. Fortunately, subsequent studies proposed various methods to construct the individual‐level brain morphological network, which has become a meaningful and reliable approach to characterizing the structural organization of the brain.^20^, ^21^, ^22^

In this paper, we mainly focus on the recent development of brain morphological networks. First, we introduced multiple methods of individual‐level morphological network construction; then we summarized the representative work, including the morphological network of normal human brain and abnormal changes of the network associated with neuropsychiatric disorders; finally, we discussed the challenges in this field and gave an outlook on possible future research directions.

## 2 INDIVIDUAL‐BASED MORPHOLOGICAL BRAIN NETWORKS

**FIGURE 2:**
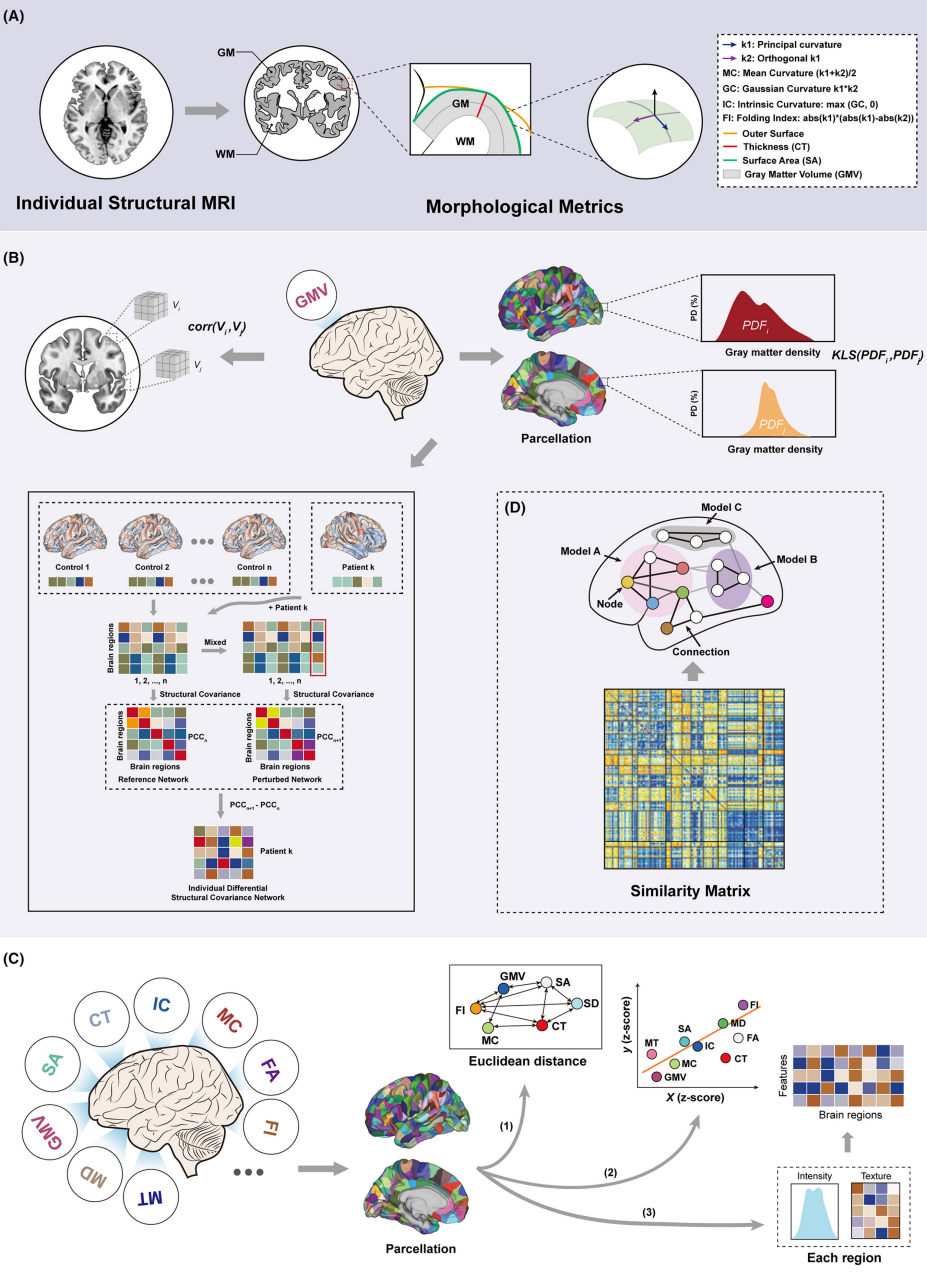
The construction of individual‐level brain morphological similarity networks. (A) The basis for constructing individual‐level morphological brain networks: morphological metrics extracted from individual structural MRI. (B) The single metric‐based methods for constructing individual morphological similarity networks, such as calculating the statistical correlation of morphological metric between different cubes^20^ (top‐left panel), estimating the similarity in the distribution of regional morphological indicator^21^, ^22^, ^27^ (top‐right panel), and the network template perturbation approach^29^ (bottom‐left panel). (C) The multiple metrics‐based methods for constructing morphological similarity networks, including (1) defining multivariate Euclidean distance to depict multiple metrics‐based interregional similarity,^31^ calculating the statistical correlation of (2) multiple morphological features (extracted from single‐modal or multimodal MRI)^30^, ^32^ or (3) radiomics features between regions.^33^ (D) Each of the above methods ultimately generates the similarity matrix, which is subsequently used to generate the network graphs for further graph‐theoretic analyses. CT, cortical thickness; FA, fractional anisotropy; FI, folding index; GC, Gaussian curvature; GM, gray matter; GMV, gray matter volume; IC, intrinsic curvature; MC, mean curvature; MD, mean diffusivity; MRI, magnetic resonance imaging; MT, magnetization transfer; PD, probability density; PDF, probability density function; SA, surface area; SD, sulcal depth; WM, white matter.

In recent years, a number of methods have emerged to construct individual‐level morphological similarity networks of the brain. Methodologically, they are classified into two categories: one category is to construct individual networks by estimating morphological similarity of a single indicator between pairs of brain regions, while the other uses a combination of multiple morphometric features to construct the network (Figure 2). In the following sections, we will describe the specific methods involved in each of the two categories separately.

### 2.1 Single metric‐based methods

In the early years, Tijms et al.^20^ first constructed individual brain morphological networks based on the similarity in GM morphology, mainly by dividing individual brain into a number of small cubes and comparing the similarity in the GM density between these cubes. With the use of graph theory,^11^, ^23^ they identified prominent small‐worldness and spatially distributed hubs in the networks. This method proved to be robust in describing individual brain morphology. However, nodes in the brain network have changed from atlas‐based regions into voxel‐based cubes, and these rigidly extracted cubes might not correspond well to anatomically or functionally specific regions of the brain.

Considering the limitation mentioned above, Kong et al.^24^ put forward a novel approach for the construction of individual brain networks based on regional morphological distribution, which offered a new perspective on brain morphology. Under this framework, nodes of the network were defined as different regions, and whole‐brain morphological connections were quantified as the statistical similarity in regional GM intensity distributions in terms of the Kullback–Leibler (KL) divergence.^25^ Likewise, the network built in such a way reflected stable topological characteristics of individual brain morphology.^21^ In the research by Wang et al.,^22^ the single‐subject morphological network was constructed using the GM volume distribution information of different regions via KL divergence‐based similarity measure.^25^ Furthermore, the long‐term test–retest reliability^26^ of this method in characterizing the morphological connectivity and topological structure of brain networks was also evaluated. As a result, the morphological brain network demonstrated specific organization with several crucial topological features (i.e., small‐world organization, high parallel efficiency, modularity, and highly connected hubs) of high test–retest reliability. Their findings indicated that the individual‐level morphological network analysis is a reliable and reproducible approach in the characterization of brain morphological organization, while future studies are needed to examine other topological properties.

Subsequently, Li and colleagues shifted the focus of their work from three‐dimensional GM volume space to two‐dimensional brain surface space where there are more metrics that can be utilized (e.g., fractal dimension, gyrification index, sulcal depth, and cortical thickness).^27^ They found that the surface‐based individual morphological similarity network exhibited nontrivial topological organization; the interregional similarity and network properties were test–retest reliable, and the morphological similarity estimated with Jensen–Shannon (JS) divergence^28^ was observed to be superior to KL divergence. This work which extended from Kong et al.^24^ brought useful insights to the characterization of human brain connectome, although its feasibility on other morphological indicators remains to be explored.

Recently, a network template perturbation approach for constructing individual structural covariance network (SCN) using regional GM volume information has been proposed by Liu et al.,^29^ and it can be used to explore the clinical and genetic correlation of structural covariance. To be specific, they constructed a reference SCN across a group of normal controls by calculating the Pearson correlation coefficients between GM volumes of each region, and then the perturbed SCNs were constructed by adding one patient to the control group at a time; the differential SCN for each patient was denoted by the difference between the perturbed network and reference network.

### 2.2 Multiple metrics‐based methods

Apart from the above methods of constructing individual‐based brain morphological networks using a single morphometric feature, researchers have also proposed several approaches to describe interregional cortical morphological similarity across multiple different metrics,^30^, ^31^, ^32^, ^33^ which can not only assess the morphological variability at an individual level but also obtain a comprehensive and systematic characterization of the morphological and organizational patterns of the brain.

The individual morphological networks based on multiple metrics can be divided into single‐modal and multimodal networks according to the use of imaging modality. The former typically uses sMRI data to extract morphometric features of brain structural organization and to calculate the interregional similarity of these metrics. For instance, Li et al.^30^ employed a Pearson correlation‐based method to estimate the similarities between seven morphometric features of different regions. In this approach, seven metrics (number of vertices, mean and Gauss curvature, surface area, GM volume, as well as mean and standard deviation of cortical thickness) of each region were concatenated as a feature vector, and the Pearson correlation coefficients between the feature vectors of any pair of regions were used to estimate interregional morphological connectivity. Afterward, Zhao et al.^33^ employed a total of 25 radiomics features (7 intensity features and 18 texture features) to characterize regional brain morphology. The single‐subject regional radiomics similarity network (R2SN) was then constructed by calculating the Pearson correlation coefficients between these radiomics features extracted from any two brain regions. The morphological networks constructed by the above‐mentioned two methods exhibited reproducible small‐world properties.^30^, ^33^

Yu et al.^34^ took another perspective by using the exponential function of multivariate Euclidean distance as an assessment of the similarity between two regions to construct a brain morphological network.^31^ The multivariate Euclidean distance was calculated based on six morphometric metrics (cortical thickness, surface area, GM volume, sulcal depth, metric distortion, and mean curvature) extracted from each vertex within each region. Small‐worldness with high reproducibility suggested the robustness of this method.

Through integrating structural MRI with other modalities, multimodal brain morphology networks can synthetically describe the morphological similarity between regions.^9^, ^35^, ^36^ Seidlitz et al.^32^ combined structural and diffusion MRI to construct a multimodal brain morphology network based on three types of morphometric features, namely cortical curvature, myelination marker, and GM morphology, aiming to provide a multi‐dimensional description of brain morphological properties. The network was specifically organized in a small‐world and modular fashion with some high‐degree hubs.

## 3 APPLICATIONS IN THE HUMAN BRAIN

Several studies presented possible biological interpretations of the brain morphological network and showed its high‐degree similarity to other forms of brain connectivity networks. The morphological covariation or similarity maybe a reflection of coordinated development or synchronized maturation between brain regions.^37^, ^38^ Seidlitz et al.^32^ demonstrated that the cytoarchitectonic classification of morphologically similar regions was more probably to be the same by aligning individual‐level brain morphological networks with classical cytoarchitectonic atlases, and this correspondence supports the biological validity of morphological similarity connectivity. Gong et al.^39^ found that interregional cortical thickness correlation across individuals may partially reflect potential fiber connectivity. These findings suggest that the connectivity of brain morphological networks may be driven by micro‐ or macro‐anatomical structures. Brain morphological networks and functional networks also exhibited similar connectivity patterns. Sun et al.^40^ reported robust correspondence of individual‐level morphological connectivity and functional connectivity, and the correspondence was linked with human cognition. In addition, the individual morphological similarity between cortical areas has been shown to be aligned with spatial expression patterns of certain important genes.^32^ A newly published study explored the consistency and diversity between individual metabolic and morphological connectivity networks, and the results indicated that cross‐modality cooperation or specialization in these two networks might imply mechanisms for achieving higher order brain functions.^41^ To sum up, the topological features of morphological networks may rely on specific anatomical, functional, and genetic bases, which correlate with cognitive functions and can provide effective biological markers for brain research. Currently, the individual morphological network approach has been widely applied in the research of normal brain and neuropsychiatric disorders (Table 1).

**TABLE 1:** A summary of individual‐level brain morphological similarity networks applications.

Applications	Study	Subject (*N*)	Modality	Metric	Network construction method and main findings
Normal brain development	Galdi et al. (2020)	Infants (105)	sMRI + dMRI	GMV + T1w/T2w + 5 DK metrics + 5 NODDI metrics	Method: Pearson's correlation Findings: Morphological similarity information was able to precisely predict chronological brain age in neonate period.
	Fenchel et al. (2020)	Infants (241)	sMRI + dMRI	CT + MC + MI + SA + 2 DTI metrics + 2 NODDI metrics	Method: Pearson's correlation Findings: The modular organization of the network corresponded spatially to known functional networks and cytoarchitectonic categories. With age, the posterior regions became more morphologically similar, while the peri‐cingulate gyrus and medial temporal lobe became more distinct. Within‐module similarity increased with age.
	Fenchel et al. (2022)	Infants (193)	sMRI + dMRI	CT + MC + MI + SA + 2 DTI metrics + 2 NODDI metrics	Method: Pearson's correlation Findings: Individual neonatal brain morphological networks can successfully predict social–emotional behaviors at 18 months of age.
Normal brain aging	Wang et al. (2022)	Adults (1427)	sMRI	CT	Method: JS divergence‐based similarity Findings: The age‐related reconfiguration was found in individual morphological networks.
AD	Tijms et al. (2013)	Patients (38) Controls (38)	sMRI	GMD	Method: Cube partition and correlation analysis Findings: Morphological networks of AD patients were characterized by more random topologies compared to normal controls.
	Tijms et al. (2014)	Early‐onset AD (95) Late‐onset AD (120)	sMRI	GMD	Method: Cube partition and correlation analysis Findings: Worse cognitive deficits were associated with more random topologies of AD morphological networks, and age of onset can modify this relationship.
MCI	Yu et al. (2018)	sMCI (86) pMCI (84) Controls (169)	sMRI	CT + SA + GMV + SD + MD + MC	Method: Multivariate Euclidean distance Findings: Individual morphological networks based on multiple features could discriminate MCI from normal controls and stable MCI from progressive MCI.
	Zhao et al. (2022)	MCI (766) AD (283) Controls (605)	sMRI	47 radiomics features	Method: Pearson's correlation Findings: With the use of R2SN, MCI patients were divided into “similar to the pattern of NCs” and “similar to the pattern of AD” subtypes.
SCD	Peng et al. (2022)	Patients (53) Controls (54)	sMRI	GMV	Method: JS divergence‐based similarity Findings: The rich‐club organization disturbances of morphological networks in SCD could be used to investigate prevention strategies at the preclinical stage of AD.
PD	Suo et al. (2021)	PD‐M (24) PD‐N (17) Controls (29)	sMRI	GMV	Method: KL divergence‐based similarity Findings: The morphological network deficits of default mode network and cerebellar characterized early PD, and frontoparietal network disruption was only associated with PD‐M, which had diagnostic potential.
Schizophrenia	Morgan et al. (2019)	Patients (185) Controls (227)	sMRI + dMRI	GMV + SA + CT + GC + MC + 2 DWI metrics	Method: Pearson's correlation Findings: Schizophrenia was associated with abnormal morphological similarity network. The combination of neuroimaging and transcriptional data provided insight into how genetic factors may drive structural brain network changes mediating the genetic risk of schizophrenia.

Zhao et al. (2020)	Patients (135) Controls (148)	sMRI	GMV	Method: KL divergence‐based similarity Findings: The combination of multiple modalities yielded the highest accuracy in classifying schizophrenic and normal individuals, suggesting that brain networks constructed based on different modalities of MRI data could provide complementary information.

Liu et al. (2021)	Patients (713) Controls (565)	sMRI	GMV	Method: Network template perturbation method Findings: Schizophrenia patients were highly variable in the changed structural covariance edges, and covariance‐based subgroups in schizophrenia were identified.
Early‐phase psychosis	Homan et al. (2019)	Patients (82) Controls (58)	sMRI	CT	Method: KL divergence‐based similarity Findings: Individual differences in brain morphological networks could predict treatment response of early‐phase psychosis.
Depression	Chen et al. (2017)	Patients (33) Controls (33)	sMRI	GMV	Method: Cube partition and correlation analysis Findings: MDD patients exhibited impaired integration and increased segregation of morphological brain networks, mainly involving neocortex‐striatum‐thalamus‐cerebellum and thalamo‐hippocampal circuits. Furthermore, MDD‐related alterations were correlated with symptom severity.
	Li, H. et al. (2021)	NSD (50) SU (50) Controls (50)	sMRI	GMV	Method: KL divergence‐based similarity Findings: Suicidality was involved in complex organization of neocortical networks. This study provided insights into the underlying neurobiology of the suicidal brain and new evidence for possible therapeutic targets for suicidal depressed patients.
	Li, J. et al. (2021)	Patients (259) Controls (243)	sMRI + dMRI	SA + CT + GMV + GC + MC + 2 DWI metrics	Method: Pearson's correlation Findings: MDD patients showed replicable alterations in morphological similarity network, and such alterations were spatially correlated with MDD‐related genes. Microglia‐ and neuron‐specific expression accounted for most of the observed correlation with MDD‐specific morphological similarity network alterations.
	Xue et al. (2023)	Patients (71) Controls (69)	sMRI	GMV + CT + SA + GC + MC	Method: Pearson's correlation Findings: Morphological similarity network gradient was significantly decreased in sensorimotor regions, and increased in visual‐related regions in MDD patients. Additionally, changes of the gradient in the left postcentral cortex and right lingual cortex showed significant correlations with symptom severity.
	Han et al. (2022)	Patients (195) Controls (78)	sMRI	GMV	Method: Network template perturbation method Findings: Depressed patients showed remarkable heterogeneity in the distribution of differential structural covariance edges, and this has helped to identify two neuroanatomical subtypes.
ADHD	Su et al. (2022)	Patients (60) Controls (60)	sMRI	GMV	Method: Multiple methods including KL divergence‐based similarity, multivariate Euclidean distance, as well as cube partition and correlation analysis. Findings: The three methods revealed different morphological connectivity patterns in ADHD. In KL divergence‐ and multivariate Euclidean distance‐based networks, ADHD exhibited weaker small‐worldness, while nodal profiles abnormalities were mainly in the corpus striatum and default mode network.
ASD	He et al. (2021)	Patients (40) Controls (38)	sMRI	GMV	Method: KL divergence‐based similarity Findings: ASD children showed poor efficiency of individual morphological brain networks. The impaired morphological connectivity patterns could predict the severity of social communication deficits in ASD children.
PTSD	Niu et al. (2018)	Patients (22) Controls (22)	sMRI	GMD	Method: Cube partition and correlation analysis Findings: The analysis of GM network topology in children with PTSD indicated a more segregated and integrated organization.

Abbreviations: AD, Alzheimer's disease; ADHD, attention‐deficit/hyperactivity disorder; ASD, autism spectrum disorder; CT, cortical thickness; DK, diffusion kurtosis; dMRI, diffusion magnetic resonance imaging; DTI, diffusion tensor imaging; GC, Gaussian curvature; GMD, gray matter density; GMV, gray matter volume; JS, Jensen–Shannon; KL, Kullback–Leibler; MC, mean curvature; MCI, mild cognitive impairment; MD, metric distortion; MDD, major depressive disorder; MI, myelin index; *N*, number; NCs, normal controls; NODDI, neurite orientation dispersion and density imaging; NSD, patient with depression and no suicidality; PD, Parkinson's disease; PD‐M, Parkinson's disease with mild cognitive impairment; PD‐N, Parkinson's disease with normal cognition; pMCI, progressive mild cognitive impairment; PTSD, post‐traumatic stress disorder; R2SN, regional radiomics similarity network; SA, surface area; SCD, subjective cognitive decline; SD, sulcal depth; sMCI, stable mild cognitive impairment; sMRI, structural magnetic resonance imaging; SU, patient with depression and suicidality; T1w, T1‐weighted signal; T2w, T2‐weighted signal.

### 3.1 Normal brain development and aging

Human brain development is a long process with dynamic alterations in its architecture across the lifespan. Revealing the changes in topological properties of brain morphological networks in the developing brain is critical for us to understand human brain development and the generation of brain functions.^42^ Galdi et al.^43^ indicated that the interregional morphological similarity information was able to precisely predict chronological brain age in neonate period, and it was helpful to investigate neuroanatomical variants associated with preterm birth. Another study of normal brain development in infants found that neonatal multimodal brain morphological networks exhibited a distinct modular distribution spatially corresponding to known functional networks and cytoarchitectonic categories.^36^ As age goes on, the posterior regions became more and more similar in morphology, while peri‐cingulate and medial temporal areas changed in the opposite direction; the similarity within modules gradually increased, suggesting emerging inter‐module differences. The neonatal morphological network was proven to be predictive of social–emotional performance in infancy as well.^44^ Besides, multifeature brain morphology network was superior to the single‐metric approach in distinguishing preterm from full‐term infants.^43^ The above studies all focus on brain development in infants, and future applications of individual‐level morphological networks in the research of brain development in other populations, including fetuses, children, and adolescents, are also valuable and noteworthy.

Aging in healthy populations is also commonly accompanied by changes in brain network topologies, leading to decreased information processing and cognitive performance. By exploring the trajectory of changes in cortical topology throughout the life cycle, Wang et al.^45^ confirmed the age‐related morphological network reconfiguration, and the findings may have implications for understanding age‐associated cognitive decline and for developing interventions to support healthy brain aging.

### 3.2 Neuropsychiatric disorders

In several common neurodegenerative diseases, researchers found abnormalities in morphological networks. There has been evidence to indicate that Alzheimer's disease (AD) is indeed a disease of dysconnectivity.^46^ By using the single‐subject GM morphological network approach, Tijms and colleagues found more random topologies of AD patients, which were associated with more severe cognitive deficits.^47^, ^48^ The morphological similarity measurement can better reveal cortical structural changes and impairments in memory as well as cognition in patients with AD. Mild cognitive impairment (MCI) is regarded as a high‐risk state for the progression to AD.^49^ Individual morphological networks based on multiple features had good classification performance in distinguishing subtypes of MCI, which facilitated the detection, risk assessment, and early precision treatment of this disease.^31^, ^50^ Alterations in morphological networks of individuals with subjective cognitive decline can also be used to study prevention strategies in the preclinical phase of AD.^51^ A study by Suo et al.^52^ showed disrupted topological organization of GM networks in the early stages of Parkinson's disease (PD). Deficits in the connectivity of default mode network and cerebellar regions were characteristics of early PD both with and without MCI, and the disruption of frontoparietal network was only associated with PD with MCI. The above findings facilitate accurate classification between PD and healthy controls, as well as between PD subgroups.

As a complex chronic psychiatric disorder, schizophrenia is accompanied by a series of psychotic symptoms and cognitive changes, which have been attributed to abnormalities of connectivity in the disorder.^53^ In 2019, Morgan et al.^54^ confirmed that the morphological similarity network phenotype was a reliable marker of cortical connectivity network abnormalities in patients with schizophrenia through analyzing three independent data sets. They also combined the imaging data with whole‐brain gene expression data from the Allen Human Brain Atlas (https://human.brain‐map.org/), and the pattern of alterations in morphological similarity was found to be spatially correlated with the expression of genes associated with schizophrenia. The combination of neuroimaging and genomics provided a more comprehensive understanding of schizophrenia, which may contribute to future treatment. In 2020, Zhao et al.^55^ constructed the functional network, anatomical (dMRI) network, and morphological network for each participant separately to investigate brain connectivity abnormalities in schizophrenia and the relationships between different imaging modalities. As a result, the accuracy of identifying schizophrenia patients was significantly increased after integrating observed features of the three networks; furthermore, the hub brain regions in networks of the three modalities varied considerably, suggesting that the brain networks constructed based on MRI data from distinct modalities had different emphases and could provide complementary information. In addition, individual morphological networks played important roles in identifying subgroups of schizophrenia and predicting individual‐level treatment response in early‐phase psychosis.^29^, ^56^

In depression, a common mental disorder, Chen et al.^57^ observed altered morphological connectivity in neocortex‐striatum‐thalamus‐cerebellum and thalamo‐hippocampal circuits, and first revealed that this alteration was related to the severity of depressive symptoms. Li et al.^58^ investigated the changes in GM morphological networks in suicidal patients with major depressive disorder, and their findings were expected to provide evidence to support clinical interventions for suicidal depressed patients. Other researchers and our group confirmed morphological similarity networks differences between patients with major depressive disorder and healthy controls, and the correlations between the case–control differences and the expression of depression‐associated genes in specific cell types, thereby linking brain morphological phenotype and gene expression level.^59^, ^60^ The individual differential SCN helped to determine neuroanatomical subtypes of depression, giving potential cues to its precision diagnosis and treatment.^61^

Individual morphological networks were applied in some studies of neuropsychiatric disorders in children as well. Su et al.^62^ found abnormal morphological connectivity patterns in attention‐deficit/hyperactivity disorder (ADHD) children using three methods of network construction, and underlined the effect of abnormal morphological network architecture on brain maturation in children with ADHD. The brain morphological networks of children with autism spectrum disorder (ASD) were shown to be less integrated than those of typically developing children.^63^ Researchers also found that morphological connectivity abnormalities could predict the severity of autistic symptoms in children with ASD. A study applied the individual morphological network approach to the topological organization of GM network in children with post‐traumatic stress disorder (PTSD).^64^ They identified a more segregated and integrated topological organization, which may be important for understanding brain maturation and architectural alterations in children after PTSD.

These works suggest that individual‐level morphological similarity networks can be instrumental in exploring the developmental and aging trajectories of normal human brain, revealing the pathophysiological features and etiological mechanisms of different neuropsychiatric disorders, and predicting the severity and prognosis.

## 4 CHALLENGES AND FUTURE DIRECTIONS

Despite over a decade of development, there are still some problems in the field of individual brain morphological networks that need to be addressed. The nodes and edges of a network can be defined in multiple ways in the construction of individual brain morphological networks. For the definition of network nodes, brain parcellation may affect topology and network characteristics of the brain,^65^, ^66^ thus, validating the proposed methods in other reputable brain atlases is an important issue in the future. The edges of morphological networks are usually defined as the statistical correlation/similarity of morphological measures^20^ (or a combination of them^30^, ^32^) between different nodes. Different metrics characterize brain morphometric features from different aspects, and for similarity measurement in the brain, the choice and optimal combination of morphological metrics remain to be explored. Additionally, different methods of calculating statistical correlations may also lead to differences in network properties.^67^ Overall, how to properly construct brain morphological networks and how to quantitatively compare the topological properties of the different networks are the priorities of future research.

In addition, existing studies on morphological similarity networks at the individual level examined only a few of the most general topological properties, while there are some other topological properties previously found in brain networks, such as hierarchy^68^ and rich‐club organization.^69^ It needs to be further determined whether more configurations exist in brain morphological similarity networks.

Lastly, although the individual‐level morphological network provides a way to measure interregional similarities in morphology, which helps us to better understand brain connectome, in‐depth studies with larger sample sizes are still needed to further reveal the role and biological meanings of morphological networks in neuroscience research.

## 5 CONCLUSION

In summary, the sMRI‐based individual‐level brain morphological similarity network enables us to analyze and understand the topology of an individual brain from a graph‐theoretic perspective, and provides a comprehensive and detailed picture of the cerebral organization individually, which can further facilitate the comprehension underlying the physiological basis of neurodevelopment and aging, cognitive performance, and neuropsychiatric disorders. In the past decade or so, methodology in the field of individual‐level brain morphological networks has developed rapidly, and the network is gradually evolving from single imaging modality/feature to multimodality/multiple features. In the future, more work is needed to address current challenges in this field and to further promote the application of individual morphological networks in brain research.

## CONFLICT OF INTEREST STATEMENT

The authors declare that they have no known competing financial interests.

## Data Availability

Data sharing not applicable to this article as no datasets were generated or analysed during the current study.
